# A novel robotic ureteral reconstruction technique for complex proximal strictures renal pelvis flap augmentation and buccal mucosal graft

**DOI:** 10.1186/s12894-025-01834-3

**Published:** 2025-07-04

**Authors:** Anh T. Nguyen, Jill C. Buckley

**Affiliations:** 1 Department of Urology, University of California, San Diego, San Diego, USA; 2 UC San Diego Health, 9444 Medical Center Drive, La Jolla, CA 92037-7897 USA

**Keywords:** Reconstructive urology, Upper tract reconstruction, Ureteral reconstruction, Buccal mucosal graft, Complex ureteroplasty

## Abstract

**Introduction:**

Complex proximal ureteral strictures can pose significant surgical challenges, including long or obliterated strictures, inability to perform primary anastomotic repairs, fixed renal pelvis, impaired vascular supply from prior surgeries, and poor healing of the proximal ureter. We describe a novel surgical technique for addressing these issues.

**Methods:**

Our technique involves a combination of ureterolysis, renal pelvis flap creation, and buccal mucosal grafting. The procedure commences with exposure of the renal pelvis, creation of a U-shaped renal pelvis flap as the posterior plate and a buccal mucosa graft as the anterior plate. The omentum is then secured to provide a vascular bed for the graft.

**Results:**

A total of 4 patients were included, with a mean age of 49 years. The median operating time was 4.08 h. The median post-operative length of stay was 1.5 days. At initial mean radiographic follow-up period of 3.2 months the success rate of the reconstruction was 100% with all patients demonstrating complete resolution of symptoms and radiographic improvement. Long-term follow-up was on average 22.3 months with sustained stability/improvement in radiographic hydronephrosis and symptoms, with no evidence of stricture recurrence. There were no donor site complications.

**Conclusion:**

This novel surgical technique, involving ureteroplasty with a renal pelvis flap augmentation and buccal mucosal graft (RPFA-BMG), proves effective for complex proximal ureteral reconstruction. It is particularly suitable for long proximal obliterated strictures that require a combination of tissue transfer techniques for successful ureteral reconstruction and achievement of physiologic drainage.

## Introduction

Reconstruction of complex ureteral strictures can pose significant challenges with patients often dealing with complications from long-term nephrostomy tubes or stents such as flank pain, renal impairment, and recurrent urinary tract infections until they are referred to a tertiary care reconstructive specialist. Traditional reconstruction techniques, including dismembered pyeloplasty, uretero-ureterostomy, and augmented anastomotic BMG ureteral reconstruction may not always be technically feasible in obliterated, long proximal strictures. This study presents a novel technique utilizing a combination of a renal pelvis flap and a buccal mucosa graft for ureteral reconstruction. We report our results highlighting surgical outcomes, complications, and post-operative recovery.

## Methods

A retrospective analysis was performed of all ureteral reconstruction cases that utilized a renal pelvis flap in combination with a buccal mucosal graft (RPFA-BMG). A total of 4 patients that were operated on in the years 2022 and 2023 were included in this study from a single-surgeon REDCap database of upper tract robotic reconstruction cases. Inclusion criteria encompassed patients aged 18 years and older with a documented history of ureteral stricture who underwent ureteral reconstruction with RPFA-BMG. Patient characteristics were examined including length and location of stricture, history of prior interventions for stricture, relevant past medical and surgical history, and postoperative follow-up and outcomes. Pre-operative evaluation is detailed in prior publications such as Fuller et al.

### Surgical technique

#### Preparation and anesthesia

All procedures are performed under general anesthesia, with patients positioned in a modified flank position. Access to the urethra is maintained with one leg in low lithotomy for female patients.

#### Access and stricture identification

**Fig. 1:**
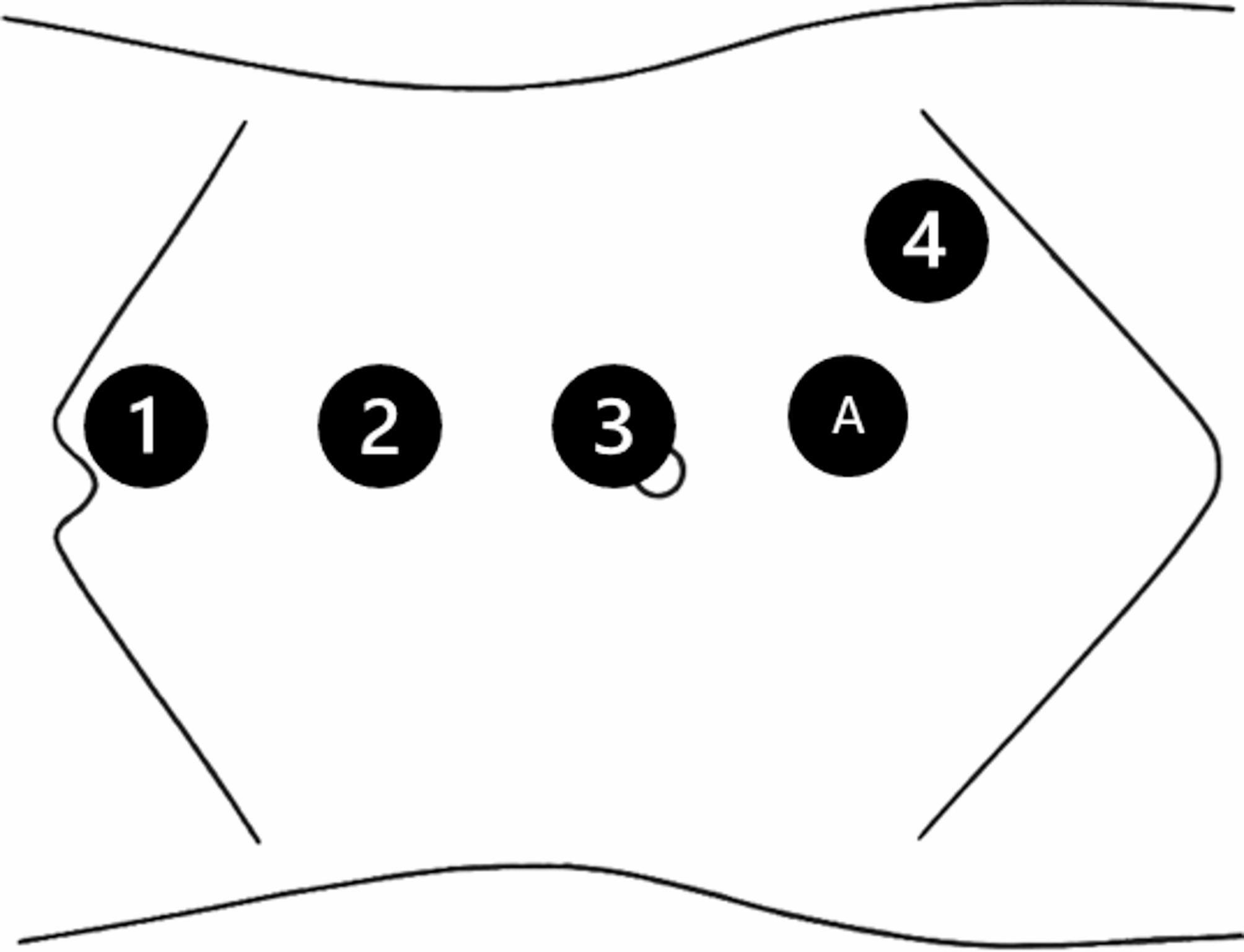
Patient positioning is in modified lateral decubitus position. A lithotomy stirrup is applied to the ipsilateral leg for female patients to allow retrograde ureteroscopic access. For male patients, the penis is prepped into the sterile field. The nephrostomy tube site if present is also prepped into the sterile field. Our typical instrument configuration is (1) Cadiere forceps, (2) Maryland bipolar, (3) Camera, (4) Monopolar scissors, and an 8 mm assistant trocar

Robotic access is established using a Veress needle in all cases and optical trocar Visiport patients with significant prior surgical history. Ureterolysis is conducted with the assistance of both antegrade and retrograde ureteroscopy to identify the stricture. Our port placement and positioning is detailed in Fig. 1.

#### Buccal mucosa graft harvesting and renal pelvis flap creation

**Fig. 2:**
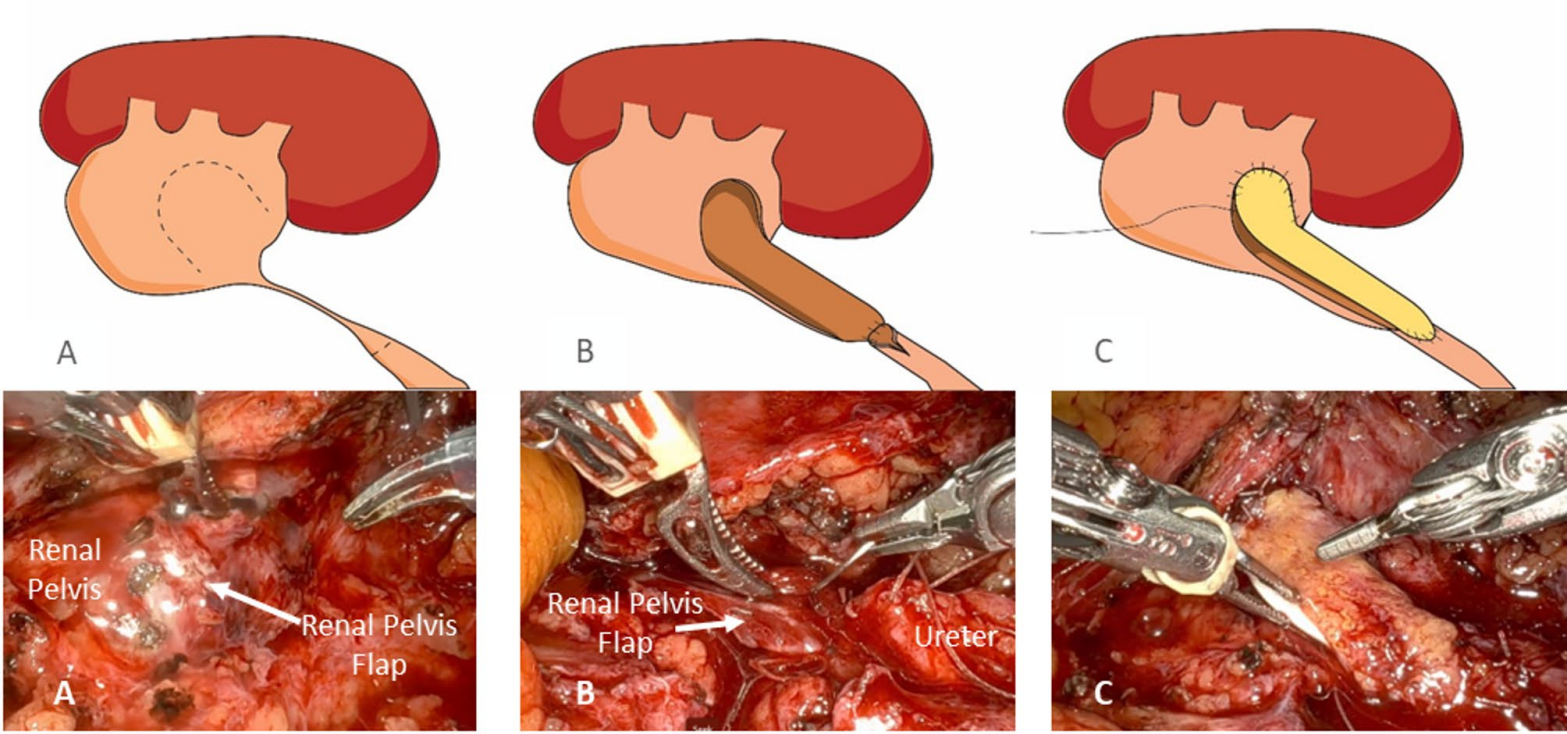
**A**. The renal pelvis flap is measured and incised. The distal ureter is transected below the level of the stricture. **B**. The renal pelvis flap is then anastomosed to the posterior plate of the distal ureter. **C**. A buccal mucosal graft is then placed as an on-lay to provide the anterior plate

A buccal mucosa graft is harvested from the inner cheek using standard technique. The typical width of graft harvested for the ureter is 1.5 cm. The harvest site is closed with absorbable sutures. A vascularized flap is created from the anterior renal pelvis and mobilized (Fig. 2).

#### Reconstruction

The renal pelvis flap is rotated to serve as the posterior plate of the ureteral reconstruction, while the buccal mucosa graft is applied as the anterior plate. The graft is secured using absorbable 5 − 0 sutures to ensure a tension-free closure. Additionally, the omentum is secured to the graft to provide a vascular bed.

#### Postoperative care

A double-J stent is placed to maintain ureteral patency and facilitate healing. Stents are routinely removed approximately 4 weeks postoperatively (2 weeks onward is acceptable). Nephrostomy tubes, if present, are removed intra-operatively. The Foley catheter is removed 5–7 days post-surgery.

### Outcome measures

Primary outcome measures included subjective improvement with resolution of symptoms (e.g., flank pain, hydronephrosis, infection) as well as objective improvement with routine renal ultrasound at 3 and 12 months postoperatively. Secondary measures encompassed postoperative complications and donor site morbidity. Data was analyzed using Excel 365. Descriptive statistics were performed, and continuous variables were expressed as mean ± standard deviation.

## Results

**Fig. 3:**
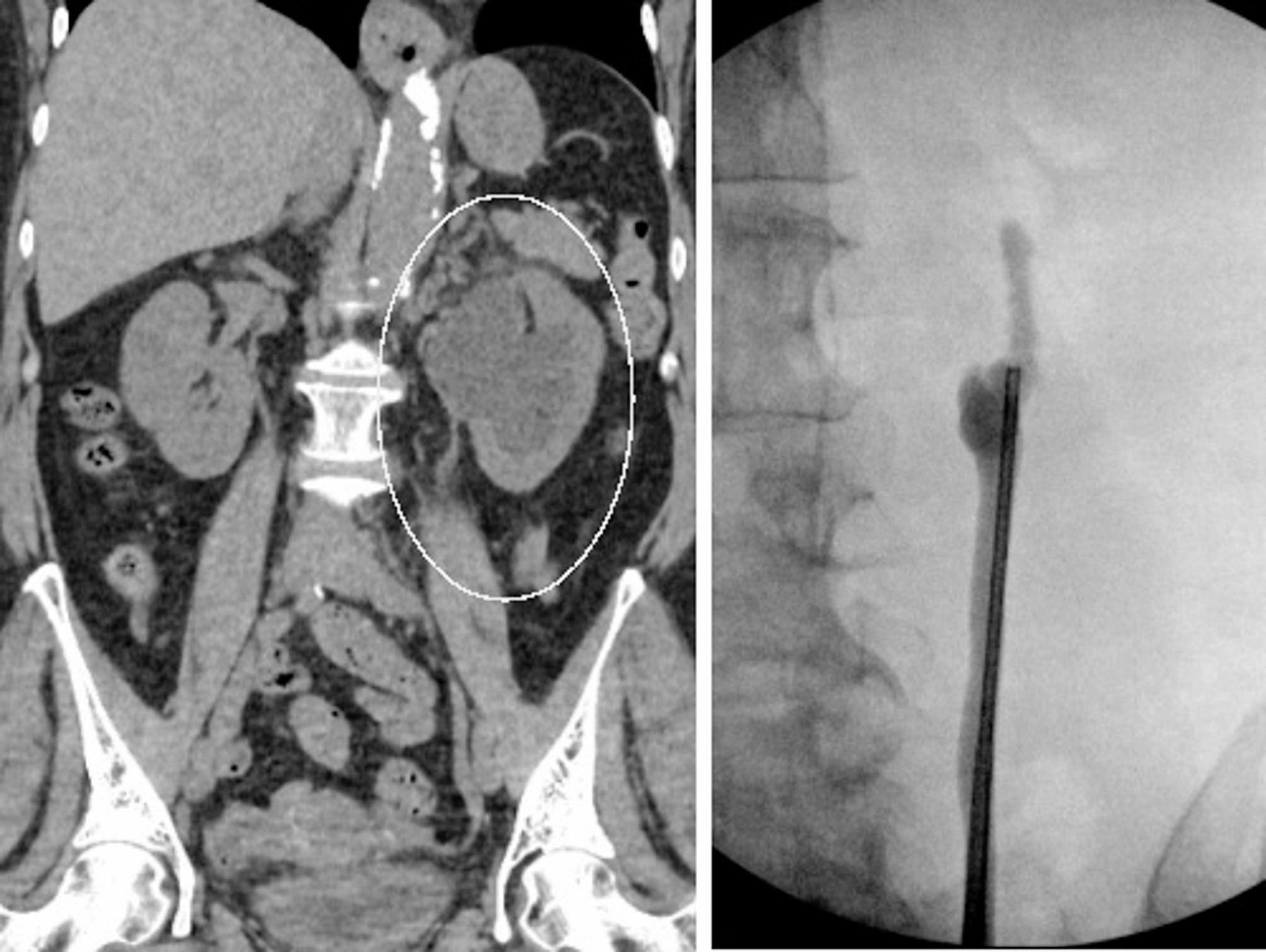
Representative computed tomography (CT) image of a patient and their associated retrograde fluoroscopic imaging study demonstrating an obliterated stricture

A total of 4 patients were included, with a mean age of 49 years (Table 1a). All four patients were married, and two were employed, one unemployed, and one retired. All four patients had insurance, with either private insurance of Medicare/Medical. The etiology for stricture was nephrolithiasis in two of the four patients. One patient had prior ureteropelvic junction repair (UPJ) for a crossing vessel and had persistent hydronephrosis and pain. One patient had a partial nephrectomy complicated by urine leak and ureteral stricture. All four patients had normal creatinine pre-operatively with a mean of 0.85 mg/dL (SD 0.28). None of the patients were active smokers, diabetic, or had prior pelvic or abdominal radiotherapy. Average BMI was 24 kg/m^2^ (SD 3). Two patients had pre-operative drainage with percutaneous nephrostomy (PCN) and one had an indwelling ureteral stent. The patient with an indwelling stent had a ureteral rest period of 21 days before reconstruction, with stent removal alone. A representative fluoroscopic and CT image is provided in Fig. 3.

**Table 1a:** demographic and operative information on patients who underwent RPFA-BMG ureteroplasty

ID	Age	Race	Etiology	Indication	Preop Drainage	Prior surgery	Operative Time (min)	EBL	LOS	Stricture Length (cm)	Obliterated
F1	39	NH White	nephrolithiasis	UTI, obstruction	nephrostomy alone	Endoscopic dilation	266	25	2	6	yes
F2	31	NH White	idiopathic	obstruction	ureteral stent	open pyeloplasty	124	55	1	3	no
M3	57	Asian	partial nephrectomy	obstruction	nephrostomy alone	none	406	200	4	2.5	yes
F4	69	H White	nephrolithiasis	obstruction	none	none	223	50	1	2	no

**Table 1b:** Post-operative complication and follow-up on patients who underwent RPFA-BMG ureteroplasty

ID	30-day complication	Preop Hydro Grade (SFU)	Time to 1st Post-op Image (months)	1st Postop Hydro Grade (SFU)	Most Recent Follow-up (months)	Latest Image Hydro (SFU)
F1	UTI	2	3.2	1	28.8	1
F2	none	3	4.4	0	22.2	0
M3	none	3	2.9	1	27.3	0
F4	none	3	2.2	0	10.8	0

All four patients had left-sided ureteral strictures with average length of 3.4 cm (SD 1.6), and 50% were obliterated (Table 1a). The median operating time was 4.08 h, with the post partial nephrectomy patient taking the longest at 6.8 h. Average estimated blood loss (EBL) was 82.5 mL (SD 68.8 mL), with the post partial nephrectomy patient having the highest EBL at 200 mL. The median post-operative length of stay was 1.5 days; the only patient staying 4 days was the complex repair post partial nephrectomy.

The indwelling ureteral stent was removed on average 46 days postoperatively (SD 3.7). Mostly out of scheduling convivence. The PCN was left in-situ in the post-partial nephrectomy patient because of the complexity and difficulty of the case.

At initial radiographic follow-up period of 3.2 months (SD 0.8), the overall success rate of the reconstruction was 100% with all patients demonstrating complete resolution of symptoms and radiographic improvement (Table 1b). Long-term follow-up was on average 22.3 months (SD 7.1), with sustained stability/improvement in radiographic hydronephrosis and symptoms, with no evidence of stricture recurrence. Our post-operative follow-up routine consists of renal ultrasound only, with reservation of nuclear scans or cross-sectional imaging for patients with refractory/recurrent clinical symptoms or un-improved hydronephrosis on ultrasound. There were no donor site complications.

One patient with a history of recurrent multi-drug resistant urinary tract infections (UTI) had an infection within 30 days of surgery which required hospitalization due to multi-drug resistant Escherichia coli. She did not have severe systemic symptoms and was discharged after one day once appropriate antibiotics were set up. There were no other reported complications within the 30- and 90-day postoperative period.

## Discussion

The combination of a renal pelvis flap and a buccal mucosa graft presents a promising solution for ureteral reconstruction, particularly in challenging cases with longer proximal and obliterated strictures where a primary repair or BMG-only alone is inadequate. This technique provides both a robust vascularized flap from the renal pelvis and simultaneous tailoring of the pelvis. The repair maintains gravity dependent drainage and a funnel shape to the renal pelvis.

Other studies have looked at using renal pelvis flaps in long proximal strictures [1]. In the pediatric literature, the use of a rotational or spiral renal pelvis flap was first presented by Culp and de Weerd in 1951 [2]. Recently, the spiral flap has been described robotically in a horseshoe kidney UPJO by Khoder et al. [3] and in an adult for secondary UPJ repair by Grice et al. [4]. In the adult population, use of buccal mucosal grafts in robotic ureteral reconstruction has been established as highly effective for proximal and/or multifocal stricture not amenable to ureteroureterostomy or standard ureteropyelostomy [5, 6]. However, in obliterated or nearly obliterated strictures, there may not be enough tissue to form an adequate lumen with just a renal pelvis flap; and a BMG alone can only augment a pre-existing lumen and cannot substitute both the anterior and posterior plate of the ureter.

In a study by the CORRUS research group [5]the techniques used were BMG onlay and/or augmented anastomotic robotic ureteroplasty with buccal mucosa. The augmented anastomotic ureteroplasty is similar in concept to the RPFA-BMG with approximation of the posterior plate of the ureter with re-anastomosis of the pelvis and ureter with the anterior plate being BMG, but our RPFA-BMG technique allow for additional length by raising a U-shaped flap from the pelvis to serve as the posterior plate. They reported median stricture length of 3.0 centimeters, 87.0% cases were surgically successful. As such, use of the renal pelvis in ureteral reconstruction is well established and we combine this concept with use of BMG in challenging strictures where a dismembered pyeloplasty, pelvis flap alone, or BMG alone is not enough to reconstruct the ureter.

In most cases of a long obliterative segment, an augmented anastomotic approach is needed where the obliterated segment of ureter is excised and a posterior plate of native ureter to native ureter is formed. The buccal mucosa is then anastomosed to the ureterotomy with omentum as a vascular backing as described previously [6]. However, in cases where a primary native ureter-to-ureter posterior plate is not feasible due to long stricture length, or immobility of the proximal or distal ureter from infection, surgical scarring or urinoma, we believe the described RPFA-BMG technique is an excellent option. This technique may obviate the need to perform extensive nephropexy or ureterocalicostomy which have their own respective complication profile. The RPFA-BMG technique would not be ideal in cases of intra-renal pelvis or excessively long strictures. In our study, the longest stricture length successfully repaired with this technique was 6 cm. All four patients in our small study describing this novel technique had excellent radiographic and clinical outcomes with resolution of hydronephrosis and removal of urinary drainage tubes. Larger cohorts and longer follow-up will validate this technique for complex obliterative and long proximal ureteral strictures.

## Conclusion

The novel ureteral reconstruction technique using a renal pelvis flap combined with a buccal graft offers a successful and durable solution for complex proximal and obliterated ureteral strictures. Our results demonstrate high success, with minimal complications that is well tolerated by patients. offering a solution for a challenging reconstructive problem. Further investigation is warranted with larger studies.

## Data Availability

The datasets used and analyzed during the current study are available from the corresponding author on reasonable request.

## References

[1] Cheng S, Li X, Yang K, Xiong S, Li Z, Zhu H, Zhang P, Li X, Guan H, Li Z, Hao H, Zhang L, Li X, Zhou L. Modified laparoscopic and robotic flap pyeloplasty for recurrent ureteropelvic junction obstruction with a long proximal ureteral stricture: the wishbone anastomosis and the ureteral plate technique. Urol Int. 2021;105(7–8):642–9. Epub 2021 Feb 10. PMID: 33567431. 33567431 10.1159/000512994

[2] Poulakis V, Witzsch U, Schultheiss D, Rathert P, Becht E. Die Geschichte der operativen Behandlung der Harnleiterabgangsstenose (Pyeloplastik). Von Trendelenburg (1886) bis zur Gegenwart [History of ureteropelvic junction obstruction repair (pyeloplasty). From Trendelenburg (1886) to the present]. Urologe A. 2004;43(12):1544-59. German. 10.1007/s00120-004-0663-x. PMID: 15316607. 10.1007/s00120-004-0663-x 15316607

[3] Khoder WY, Alghamdi A, Schulz T, Becker AJ, Schlenker B, Stief CG. An innovative technique of robotic-assisted/laparoscopic re-pyeloplasty in horseshoe kidney in patients with failed previous pyeloplasty for ureteropelvic junction obstruction. Surg Endosc. 2016;30(9):4124–9. 10.1007/s00464-015-4678-8. Epub 2015 Dec 16. PMID: 26675936. 26675936 10.1007/s00464-015-4678-8

[4] Peter T, Grice, Henry HI, Yao J. Walton. Robotic culp de weerd pyeloplasty for secondary pelviureteral junction obstruction. Videourology 2021 35:2. 10.1089/vid.2020.0090

[5] Lee Z, Lee M, Koster H, Lee R, Cheng N, Jun M, Slawin J, Zhao LC, Stifelman MD, Eun DD. Collaborative of reconstructive robotic ureteral surgery (CORRUS). A Multi-Institutional experience with robotic ureteroplasty with buccal mucosa graft: an updated analysis of Intermediate-Term outcomes. Urology. 2021;147:306–10. Epub 2020 Aug 13. PMID: 32798516. 32798516 10.1016/j.urology.2020.08.003

[6] Fuller TW, Daily AM, Buckley JC. Robotic ureteral reconstruction. Urol Clin North Am. 2022;49(3):495–505. 10.1016/j.ucl.2022.05.002. Epub 2022 Jun 27. PMID: 35931439. 35931439 10.1016/j.ucl.2022.05.002

